# Facing the Unknown: Integration of Skeletal Traits, Genetic Information and Forensic Facial Approximation

**DOI:** 10.3390/genes16050511

**Published:** 2025-04-28

**Authors:** Joe Adserias-Garriga, Francisco Medina-Paz, Jorge Molina, Sara C. Zapico

**Affiliations:** 1 Department of Applied Forensic Sciences, Mercyhurst University, 501 East 38th Street, Erie, PA 16546, USA; jadseriasgarriga@mercyhurst.edu; 2 Department of Chemistry and Environmental Science, New Jersey Institute of Technology, 161 Warren St., Newark, NJ 07102, USA; francisco.medinapaz@njit.edu; 3 Texas Department of Public Safety, 5805 North Lamar Blvd, Austin, TX 78752, USA; jorge.molina@dps.texas.gov; 4 Anthropology Department and Laboratories of Analytical Biology, National Museum of Natural History, Smithsonian Institution, 10th and Constitute Ave, NW, Washington, DC 20560, USA

**Keywords:** biological profile, facial approximation, DNA phenotyping, integration

## Abstract

Background/Objectives: Identification of human remains is of utmost importance for criminal investigations and providing closure to the families. The reconstruction of a biological profile of the individual will narrow down the list of candidates for identification. From another perspective, facial approximations performed by a forensic artist can provide investigative leads, with the identity being confirmed by primary or secondary methods of identification. In recent years, DNA analysis has evolved, trying to create a portrait of the perpetrator/victim based on External Visible Characteristics (EVCs), the color of the eyes, hair, and skin and Biogeographical ancestry (BGA), called DNA phenotyping. Despite these advances, currently, there are no studies integrating the biological profile performed by forensic anthropologists, the facial approximation created by forensic artists and EVCs determined by DNA. The goal of this work was to integrate these three investigative leads to enhance the possibility of human identification. Methods: Five donated remains from Mercyhurst were studied through these approaches: reconstruction of biological profile, facial approximation and estimation of EVCs based on previous studies. Results: Our results indicated the feasibility of integrating this biological profile and EVCs data into the facial approximation developed by the forensic artist, aiming to an enhance portrait of the remains. In a second phase of this project, the accuracy of the integrated facial approximation will be assessed. Conclusions: This study pointed out the importance of an interdisciplinary approach towards the identification of human remains, as well as the combination of current methods with new technologies.

## 1. Introduction

When human remains are found, the first step in the identification process is to reconstruct the biological profile of the individual, which consists of a broad description of the deceased regarding population affinity, biological sex, age and stature. The reconstruction of the biological profile of the skeleton is one of the pillars of forensic anthropology and provides critical information in the identification of unknown human remains. Each component (population affinity, biological sex, age and stature) relies on established morphological and/or metric methods. From all the biological profile components, biological sex and age are the most relevant for narrowing down the list of candidates for the identity.

Estimation of population affinity remains one of the most challenging aspects of the biological profile. Hefner’s method for population affinity estimation [1] as well as Hefner and Ousley [2] rely on a combination of nonmetric cranial traits and discriminant function analysis. Hefner’s approach uses a probabilistic framework to classify individuals into populational groups based on cranial traits. Fordisc, a software program developed by Ousley and Jantz [3], is another widely used tool for population affinity estimation. Fordisc employs discriminant function analysis to classify individuals based on cranial measurements (although postcranial measurements can be used, they generally are not very informative). The software allows forensic anthropologists to compare an unknown individual’s measurements to these reference groups, providing a statistical probability of group membership. While Fordisc is a powerful tool, its accuracy depends on the availability of population-specific reference data and the appropriateness of the comparative samples.

Biological sex estimation is most accurately determined by the os coxae, namely the ventral arc, subpubic concavity and medial aspect of the ischiopubic ramus, as described by Phenice [4]. Klales et al. [5] reported a revised scoring system for these traits that improved reliability and reduced observer error. Cranial morphology also provides informative sex indicators, including the nuchal crest, mastoid process and supraorbital margin. Walker [6] developed a probabilistic approach to sex estimation from cranial features that has been tested and validated on several populations. MorphoPASSE [7], is a computer program for sex estimation relying of the scoring of pelvic and cranial traits for sex estimation, which is widely used nowadays.

Different methods are used to estimate age-at-death for juveniles and adults. Age estimation methods for juveniles rely on growth and developmental changes in bones and teeth. Whilst age estimation in adults relies on degenerative changes in the skeleton. Most widely used adult age estimation methods are the Suchey–Brooks method of pubic symphysis [8] and the Lovejoy et al. [9] system for the auricular surface. Several authors have explored the use of cranial sutures obliteration for age estimation [10,11]. However, all methods utilizing cranial sutures closure have shown a poor correlation with chronological age [12]. İşcan et al. [13,14] developed a phase-based system that evaluates changes in the costal cartilage surface at the sternal end of the 4th rib. This method has been validated in multiple populations and is particularly useful for its precision in middle-aged and older adults. Hartnett [15,16] introduced revised standards for estimating age from the pubic symphysis and sternal rib ends that provide increased accuracy and restrict interobserver error.

Overall, the biological profile of the skeleton remains a vital component in forensic anthropology. While traditional methods are a good basis, ongoing research and technological advancement continue to improve their precision and applicability. The integration of anthropological analysis with other methods of identification such as genetic analysis and forensic art techniques could help overcome the current limitations of each group of techniques individually.

Another traditional method often employed to assist with the identification of human remains is forensic art. Forensic art is a broad and diverse discipline offering a wide range of forensic techniques to assist with suspect and victim identification in a law enforcement context. Its primary focus is to generate investigative leads by triggering recognition through the creation of facial images which enable other forensic disciplines, such as DNA analysis, to make legally valid identifications [17].

Forensic facial approximation is a forensic art technique that assists identification efforts by simulating the in-life appearance of an unidentified decedent. This is a highly collaborative effort where the forensic artist works in tandem with other forensic professionals such as pathologists, forensic anthropologists and law enforcement investigators who provide information on biological sex, population affinity and cause and/or manner of death. It is based on a procedure of approximating the soft tissue contours of the face using statistical tissue depth data. The morphological information of the skull provides the underlying architecture of the development of the soft tissue features. These facial approximations can be developed in several ways including two-dimensional and three-dimensional formats.

While forensic facial approximation is not a conclusive means of identification, it serves as a means of expediting the investigative process while investigators await more conclusive determinations of identity. The forensic facial approximation process may serve as a means of generating leads when other forensic avenues of identification have been exhausted. Its function is to generate leads rather than serve as the primary means of identification.

As one of the other primary methods of identification, DNA analysis from human remains could be crucial in cases of missing persons and disaster victim identification [18]. As a resilient molecule, DNA degrades gradually in hard tissues, such as bones and teeth, with extraction of the DNA being possible under favorable conditions [19]. However, environmental factors could affect the remains, leading to problems of degradation, inhibition and contamination of DNA and hampering the possibility of obtaining a STR profile [20]. Additionally, as more time passes since the disaster or when the person went missing, it is possible that less ante-mortem samples from relatives or the person itself would be available [21]. In these cases, other means of identification are required. Single Nucleotide Polymorphisms (SNPs) have been emerged as potential solutions to these problems [22]. SNPs are nucleotide substitutions, insertions or deletions that are normally biallelic with low mutation rates and high heritability [23,24,25]. Moreover, the small size of their PCR amplicons makes them useful to analyze in cases of degraded or scarce DNA. Based on them, it has been possible to develop assays for the prediction of Externally Visible Characteristics (EVCs) and biogeographical ancestry (BGA) [26,27], referred to as forensic DNA phenotyping (FDP). As a result, FDP could be also useful in missing persons’ investigations and in the identification of human remains [28].

In this study, we chose to apply the HIrisPlex-S system for the prediction of eye, hair and skin color [26]. It consists of 41 DNA SNPs: 24 included in the original HIrisPlex assay and 17 SNPs investigated in another analysis to complement the skin color. It was carried out based on this original protocol, with SNaPshot™ (Single Based Extension (SBE) and Capillary Electrophoresis (CE)). The results of the analysis were introduced in the open-source software available at https://hirisplex.Erasmusmc.nl/ (accessed on 25 April 2025) to evaluate the prediction probabilities for three iris colors, four hair colors and five skin color categories [26].

These three techniques independently showed different successes in aiding with the identification of human remains, providing investigative leads. However, currently, there are no studies assessing the combination of these methods. Overall, the aim of this study was to integrate a forensic anthropology biological profile, facial approximation by a forensic artist and forensic DNA phenotyping to improve human identification.

## 2. Methods and Materials

### 2.1. Samples

Five skeletons from the Donated Collection of the Department of Applied Forensic Sciences at Mercyhurst University were included in this study.

### 2.2. Anthropological Analysis

Population affinity, biological sex and age-at-death estimations were conducted for the five individuals included in this project.

Non-metric population affinity was estimated using six macromorphoscopic traits described in Hefner [1] and the optimized summed scored attributes (OSSAs) method outlined in Hefner and Ousley [2]. Metric analysis for population affinity estimation was conducted using twenty-six standard cranial measurements recorded from an individual which were compared to individuals of known sex and ancestry through linear discriminant function analysis in FORDISC 3.1 [2,3] following the guidelines outlined in Ousley and Jantz [29].

Non-metric sex estimation was based on features of the os coxae and cranium, using ordinal scores of the 12 variables (2 unilateral and 2 binary skull traits, as well as 3 bilateral pelvis traits) and the random forest classification provided by MorphoPASSE [7].

For age estimation, the pubic symphysis, auricular surface and cranial sutures were analyzed using the computer-based age estimation program ADBOU, which utilizes transition analysis [30,31].

Stature (though not related to the facial approximation) was estimated using FORDISC 3.1 [2,3].

### 2.3. Bone Samples for DNA Analysis

Bone fragments of approximately 2 × 2 cm were collected from the femoral diaphysis for the five individuals using a Stryker 810 Autopsy Saw (Stryker, Portage, MI, USA).

### 2.4. Facial Approximation

The process of developing forensic facial approximations for the five unknown skeletal samples was initiated by three-dimensionally scanning the remains and generating a virtual 3-D model of each of the skulls. The scans contained both the mesh and texture data of the skulls facilitating the creation of virtual replicas of the skulls using a 3-D modeling application (Autodesk 3ds Max).

Multiple tissue depth data sets were then researched based on the sample profiles to ensure the most accurate soft tissue approximations. The Rhine and Moore tissue data sets for normal male and female, European-derived, samples were selected for this study. Appropriate tissue depth markers were created and placed at various craniometric landmarks on each of the skulls based on Karen Taylor’s two-dimensional facial reconstruction techniques [32].

The skulls were then aligned in the Frankfort Horizontal Plane to minimize perspective distortion. Frontal and lateral 2-D images were rendered and exported in a lossless format (.PNG). A scale was generated to facilitate accurate scaling of exported images in a digital imaging application (Adobe Photoshop).

The frontal and lateral images were aligned to correspond to one another to facilitate the development of two-dimensional frontal and lateral facial approximations in concurrent fashion. Empirical data from the skull was gathered to locate phenotypic traits on the splanchnocranium. The lateral projection of the nose was calculated using data based on the anterior nasal spine and Dr. Robert George’s technique for lateral projection [33].

### 2.5. DNA Isolation from Bone Samples

A diamond cutting disk was used to cut femur bones samples into smaller pieces. Then, the bone pieces were mechanically ground using an agate mortar and pestle and were divided into aliquots of approximately 200 mg each. DNA was isolated from the bone aliquots by using the DNeasy Blood and Tissue Kit (Qiagen, Hilden, Germany) with some modifications to the manufacturer’s instructions. First, 180 μL of ATL buffer plus 20 μL of proteinase K were added to each sample and they were mixed thoroughly by vortex. The resulting solution was then incubated (overnight) under agitation at 56 °C until the tissue was completely lysated. The following day, 200 μL of AL buffer was added and mixed by vortexing. Then, 200 μL ethanol (100%) was added to the solution and mixed again by vortexing. The solution was transferred to a DNeasy Mini Spin column placed in a 2 mL collection tube and centrifuged at 6000× *g* for 1 min. The flow-through was discarded, and the column was placed in a new 2 mL collection tube. Next, 500 μL of AW1 Buffer was added, and the column was centrifuged at 6000× *g* for 1 min. The flow-through was discarded, and the column was placed in a new 2 mL collection tube. Later, 500 μL of AW2 buffer was added, and the column was centrifuged at 20,000× *g* for 3 min. The flow-through was discarded and the column was centrifuged one more time at 20,000× *g* for 1 min to eliminate the ethanol residues. The flow-through was discarded, and the column was placed in a new 1.5 mL tube. Then, 35 microliters of AE buffer were added directly onto the DNeasy column’s membrane and incubated for 1 min at room temperature. Finally, the column was centrifuged at 6000× *g* for 1 min to elute the DNA. The resulting DNA samples were stored at −20 °C.

As preventive measures to avoid DNA contamination of the samples, the whole molecular biology workflow, from DNA extraction through sequencing, was performed under sterile working conditions: working under a biological safety cabinet II UV-sterilized before and after being used, utilizing new sterile material such as pipette tips with filters, nitrile gloves discarded after one single use and utilizing molecular-grade sterile water.

### 2.6. DNA Quantification

The DNA samples were quantified by using the Qubit dsDNA HS Assay Kit (Invitrogen, Life Technologies, Carlsbad, CA, USA), according to the manufacturer’s protocol.

### 2.7. Hair and Eye Color Genotyping System Protocol

For the genotyping of hair and eye color, we used the HIrisPlex system. The HIrisPlex system is based on the evaluation of 23 SNPs and 1 insertion/deletion (INDEL) polymorphism from 11 genes [34]. All the marker details, primer sequences, and concentrations are provided in Table 1. PCR amplification of all SNPs was performed in a single multiplex PCR assay with a total volume of 12 μL containing PCR primers, concentrations described in Table 1, 1 μL genomic DNA isolated from bones (1 ng/μL), 1X PCR buffer (Applied Biosystems. Waltham, MA, USA), 2.7 mM MgCl_2_ (Applied Biosystems), 200 μM of each dNTP (Promega Corporation. Madison, WI, USA) and 0.5 U AmpliTaq Gold 360 DNA Polymerase (Applied Biosystems). The thermocycler PCR parameters were set as follows: 95 °C for 10 min; 33 cycles of 95 °C for 30 s, 56 °C for 30 s, 72 °C for 30 s and a final elongation phase of 72 °C for 7 min. The PCR products were purified using ExoSAP-IT Express (Applied Biosystems) and incubated at 37 °C for 4 min and 80 °C for 1 min. The multiplex SBE (single base extension) was carried out for all 24 products at the same time in a single multiplex reaction using 2 μL of the purified PCR product, 2 μL of the SBE primer mix (final concentrations of each SBE primer in Table 1) and 1 μL of the ABI Prism^®^ SNaPshot Multiplex Kit (Applied Biosystems) with the following thermocycler parameters: 96 °C for 2 min; 25 cycles of 96 °C for 10 s, 50 °C for 5 s and 60 °C for 30 s. The resulting products were purified using 1 μL of shrimp alkaline phosphatase (SAP) (Applied Biosystems) and incubated 37 °C for 45 min and 75 °C for 15 min. Finally, the purified SBE products were analyzed on the SeqStudio Genetic Analyzer machine (Applied Biosystems) with Applied Biosystems POP-1 polymer on a 28 cm capillary length under an injection voltage of 1.2 kV for 7 s and with a running time of 330 s at 60 °C. Gene Mapper ID-X v1.6 software (Thermo Fisher Scientific, Waltham, MA, USA) was used for analysis of the results.

### 2.8. Skin Color Genotyping System Protocol

For the genotyping assay of skin color, the HIrisPlex-S (HPS) system was used following Chaitanya et al.’s [26] protocol, a 17-plex system involving the evaluation of 36 SNPs from 16 genes for skin color. A detailed mention of all the markers, primer sequences, and concentrations is provided in Table 2. PCR amplification of all SNPs was performed in a single multiplex PCR assay with a total volume of 12 μL containing PCR primers, concentrations described in Table 2, 1 μL genomic DNA isolated from bones (1 ng/μL), 1X PCR buffer (Applied Biosystems. Waltham, MA, USA), 2.7 mM MgCl_2_ (Applied Biosystems), 200 μM of each dNTP (Promega Corporation, Madison, WI, USA) and 0.5 U AmpliTaq Gold 360 DNA Polymerase (Applied Biosystems). The thermocycler PCR parameters were set as follows: 95 °C for 10 min; 33 cycles of 95 °C for 30 s, 55 °C for 30 s, 72 °C for 30 s and a final elongation phase of 72 °C for 7 min. The PCR products were purified using ExoSAP-IT Express (Applied Biosystems) and incubated at 37 °C for 4 min and 80 °C for 1 min. The multiplex SBE (single base extension) was carried out for all 17 products at the same time in a single multiplex reaction using 2 μL of the purified PCR product, 2 μL of the SBE primer mix (Table 2) and 1 μL of the ABI Prism^®^ SNaPshot Multiplex Kit (Applied Biosystems) with the following thermocycler parameters: 96 °C for 2 min; 25 cycles of 96 °C for 10 s, 50 °C for 5 s and 60 °C for 30 s. The resulting products were purified using 1 μL of shrimp alkaline phosphatase (SAP) (Applied Biosystems) and incubated 37 °C for 45 min and 75 °C for 15 min. Finally, the purified SBE products were analyzed on the SeqStudio Genetic Analyzer machine (Applied Biosystems) with Applied Biosystems POP-1 polymer on a 28 cm capillary length under an injection voltage of 1.2 kV for 7 s and with a running time of 330 s at 60 °C. Gene Mapper ID-X v1.6 software program (Thermo Fisher Scientific, Waltham, MA, USA) was used for analysis of the results.

### 2.9. Hair, Eye and Skin Color Prediction

The prediction of eye, hair and skin color from the donors was performed based on the identification of the specific SNPs described in previous sections and populated on the open source website reported by Chaitanya et al. [26], known as HIrisplex-S: HIrisPlex-S Eye, Hair and Skin Colour DNA Phenotyping Webtool: https://hirisplex.Erasmusmc.nl/. This tool provided the probabilities of the predictive phenotypes based on the different categories of eye color, hair color and shade and skin color.

## 3. Results

### 3.1. Biological Profile

The results obtained from the skeletal biological profile estimations are summarized in Table 3, comprising population affinity, biological sex and age-at-death for the five individuals (stature was not used for facial approximation). Known data from the individuals is also included.

Individual 1’s skeletal profile estimation corresponded to a white male between 35 and 75 years of age at the time of death and between 165 and 185 cm in stature, which was consistent with the known data of the individual (white male, 66 years of age, and 170 cm). Individual 2’s skeletal profile estimation corresponded to a white male over 50 years and between 165 and 185 cm in stature, which was consistent with the known data of the individual (white male, 88 years of age and 165 cm). Individual 3 corresponded to a white male between 30 and 75 years, and 162 to 185 cm in stature, which was consistent with the known data of the individual (white male, 42 years of age and 165 cm). Individual 4’s skeletal profile estimation corresponded to a white male between 30 and 65 years, which was consistent with the known data of the individual (white male, 66 years of age and 185 cm). Individual 5’s skeletal profile estimation corresponded to a white female between 45 and 85 years of age at the time of death, which was consistent with the known data of the individual (white female, 62 years of age and 162 cm) (Table 3).

### 3.2. Phenotyping Predictions

A summary of the results from the HIrisPlex-S online tool is depicted in Table 4, including the prediction *p*-values. Regarding the eye color, the prediction was performed by comparing the probabilities obtained by entering our genotyping results into the online tool and matching the resulting probabilities to the pictures in the figures provided by Walsh et al. (2011) [35]. Hair color prediction was performed according to the recommendations from Walsh et al. (2013) based on the hair color and shade probabilities as derived from the HIrisPlex-S online tool [34]. In brief, the process for predicting the hair color and shade with the best probability of matching the true hair color is as follows: (1) Calling the category of the leading color (black, brown, red, redhead and blonde) prediction based on the highest probability value; (2) calling the final color prediction based on the contribution and effect of black and blonde using the probability values for dark and light attributes. For a clearer reference on the color and tone of the hair, we refer to the images shown in the same article [34]. Finally, skin color was predicted as described in Chaitanya et al. (2018) [26]. In their guide for skin color prediction, the authors set different threshold values for the probabilities obtained by using the HIrisPlex-S DNA test system and classify skin color in five different categories: very pale, pale, intermediate, dark, and dark to black. The pictures of the exemplified performance of their predictive model were also used in order to clarify the skin color of our samples.

According to the genotyping analysis, Individual 1 would have blue eyes and light red hair with pale skin tone. Individual 2 would have brown eyes, dark red hair and intermediate to pale skin tone. Individual 3 would have brown eyes, intermediate red hair and intermediate skin tone. Individual 4 would have blue eyes, light red hair and pale skin. Individual 5 would have blue eyes, dark red hair and pale skin tone. These phenotyping predictions have been included in the facial approximation based on the pictures displayed in previous publications [26,36].

### 3.3. Facial Approximation Integration

Using the forensic anthropological profile, 3-D skull model and accepted forensic art techniques, 2-D frontal and lateral facial approximations were developed for each of the samples in a monochromatic grayscale. These grayscale versions served as templates for developing the final facial approximation likenesses in full color using the forensic DNA phenotyping predictions for hair, eye and skin color.

**Figure 1:**
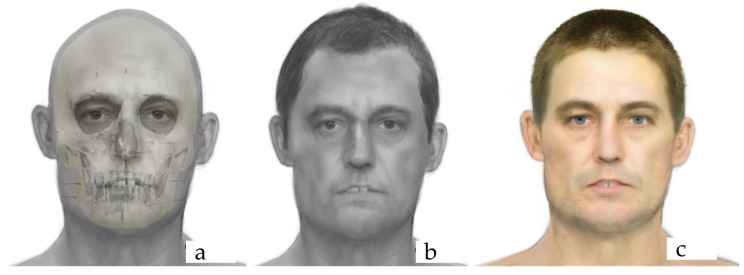
Facial approximation products of Individual 4: overlay of the soft tissues over the skull (**a**), final facial approximation with the skeletal profile estimation information (**b**), and final facial approximation with the skeletal profile estimation and phenotyping information (**c**).

Digital imaging techniques were used to enhance the final facial approximations. Additionally, superimpositions of the skulls over the facial approximations were created to illustrate the correspondence between the contours of the face and the distal points of the tissue depth markers. Figure 1 presents the frontal view of the facial approximation products.

## 4. Discussion

This technical report presented, for the first time, the application of an interdisciplinary approach, integrating a forensic anthropology biological profile, facial approximation and forensic DNA phenotyping, with the aim of improving human identification.

The next step of this project will be the comparison of the facial approximations obtained (only with the anthropological information and with the anthropological and phenotyping information) with in-life photos of the sample decedents. Therefore, the facial approximations will be assessed in terms of overall likeness and accuracy of facial proportion.

Image comparison is a topic that has been adjudicated in legal contexts in a variety of cases. However, image comparison as it relates to forensic facial approximation is not typically subject to legal scrutiny. It is a forgone conclusion that forensic facial approximations capture an approximate likeness at best as the name would suggest. Therefore, evaluating the efficacy of facial approximation in even the broadest statistical terms can prove quite challenging. Facial approximations are subject to subtle interpretations of appearance and likeness and do not necessarily fit into the prescribed standards of one-to-one image comparison used in facial recognition algorithms and facial identification analyses.

Moreover, each technique by itself has its limitations. Forensic anthropology, in the first phase of the identification process, through the estimation of the biological profile (population affinity, biological sex, age at death and stature), as well as other unique characteristics (i.e., previous ante-mortem trauma), could help to reduce the number of missing persons candidates for the identity [37]. However, if there are no other ante-mortem data like dental records or DNA reference samples from the deceased or relatives, the identification is challenging. Facial approximation could help in these cases; although it is not a scientific method of identification, it can provide visibility, especially for cold cases, and it can assist in achieving a positive identification through scientific means.

Evaluating the efficacy of forensic facial approximations through feature interpretation is inherently grounded in a qualitative analysis of likeness best expressed in terms of language rather than numerical values. Consequently, the evaluation of a facial approximation remains a nuanced process that demands a holistic appreciation for the intricacies of facial feature interpretation and its qualitative dimensions [17]. Some “successful” forensic facial approximations have elements of “built-in ambiguity” [32]. This tendency is often preferred in situations where the specificity of an image may prove detrimental to the identification process where potential leads are ruled out. Assessing likeness through an image comparison then becomes a discussion of qualitative data versus quantitative data where the results are more aptly expressed through language and value judgments instead of numerical values [17]. As mentioned previously in this article, the evaluation of the facial approximations recognition of the method presented will be assessed in the second phase of this project.

This study is based the FDP on the HIrisPlex-S system [26,34,35,36,38,39], which is the most common one used in several studies [20,37,40,41,42] applying different methodologies with advantages and disadvantages. Many of these previous works have been focused on translating these HIrisPlex-S into Next Generation Sequencing (NGS) platforms [41,43,44,45,46]. Additionally, there are tools, like VISAGE Basic Tool (BT) from the VISAGE Consortium, which includes ancestry SNPs as well as the 41 SNPs from the HIrisPlex-S panel [47]. Moreover, there are commercial solutions like the MiSeq FGx™ Forensic Genomics System, from Qiagen, with a first panel including 27 autosomal, 7 X- and 24 Y-chromosomal STRs and 94 identity-SNPs; a second panel includes 56 ancestry SNPs and 22 phenotype-informative SNPs (for eye and hair color) [22]. Parabon Nanolabs also offers the Snapshot™ DNA Phenotyping Service, creating a complete profile, including genetic ancestry, eye, hair and skin color, freckling and face shape [22]. Overall, the aforementioned platforms point out one of the main limitations of FDP: the lack of standardization of methodologies. As described, current efforts are focused on translating these panels into NGS, as they allow higher throughput, multiplexing capacity and sequencing accuracy, as well as the possibility to automate and sequence different markers in the same run [48]. However, the overall cost is too high and may not be affordable for routine use by the forensic labs [20]. As a result, the gold-standard technique is still SNaPshot™ (SBE-CE assay) due to its robustness, simplicity and efficiency, but more precisely, because the instrument is already present in forensic laboratories. This is the reason why this study applied this classical methodology instead of NGS. The idea was to develop a protocol that is affordable and accessible for forensic laboratories, using the equipment they already have available. However, it is not exempt from drawbacks; SNaPshot™ has higher risk of contamination and error, and more importantly, is limited to analyzing three single traits with 30 to 40 markers (eye, hair and skin color) [43]. Despite this, previous studies and our present work demonstrated its applicability to deteriorated DNA [49,50]. Finally, it is worth noting that, overall, these previous works, both applying classical SNaPshot™ and NGS sequencing technologies, based their facial approximation only on forensic DNA phenotyping; the present study is the first one integrating these FDP traits into facial approximation and anthropological findings, enhancing human identification.

## 5. Conclusions

This is the first interdisciplinary study integrating anthropological biological profiling, facial approximation and forensic DNA phenotyping. The findings from this research indicate the possibility of performing forensic DNA phenotyping in a forensic lab, with the instrumentation used for STR profiling in degraded human remains, and work alongside the forensic artist and forensic anthropologist to enhance facial approximation and improve human identification. Although the integrated methodology is presented in this technical report, the second phase of this project will involve an assessment of the recognition of the facial approximations with and without the information obtained from the phenotyping analysis.

Further research is needed in this new multidisciplinary approach, including more individuals in the sample encompassing wider ranges of age and including different populations.

## Figures and Tables

**Table 1:** Information about the 24 DNA variants of the HIrisPlex assay, including PCR and single base extension (SBE) primer sequences and concentrations. Major and minor alleles correspond to the input information entered to the HIrisPlex prediction model.

Assay Position	PCR Primers	Sequence	Concentration	Product Size	SBE Primers	Gene	HIrisPlex Model Input	Sequence	Concentration
1	MC1Rset1F	GCAGGGATCCCAGAGAAGAC	0.55 μm	117 bp	N29insA	MC1R	C/insA	CCCCAGCTGGGGCTGGCTGCCAA	1.3 μm
2	MC1Rset1R	TCAGAGATGGACACCTCCAG	0.55 μm		rs11547464	MC1R	G/A	ttttttttttttGCCATCGCCGTGGACC	0.1 μm
3	MC1Rset2F	CTGGTGAGCTTGGTGGAGA	0.5 μm	158 bp	rs885479	MC1R	C/T	ttttttttttttttttttGATGGCCGCAACGGCT	1.25 μm
4	MC1Rset2F	TCCAGCAGGAGGATGACG	0.5 μm		rs1805008	MC1R	C/T	tttttttttttttACAGCATCGTGACCCTGCCG	0.375 μm
5	MC1Rset3F	GTCCAGCCTCTGCTTCCTG	0.5 μm	147 bp	rs1805005	MC1R	G/T	tttttttttttttttTGGTGGAGAACGCGCTGGTG	0.75 μm
6	MC1Rset3R	AGCGTGCTGAAGACGACAC	0.5 μm		rs1805006	MC1R	C/A	ttttttttttttttttttttCTGCCTGGCCTTGTCGGA	0.75 μm
7	MC1Rset4F	CAAGAACTTCAACCTCTTTCTCG	0.4 μm	106 bp	rs1805007	MC1R	C/T	tttttttttttttttttttttttttCTCCATCTTCTACGCACTG	1 μm
8	MC1Rset4R	CACCTCCTTGAGCGTCCTG	0.4 μm		rs1805009	MC1R	G/C	ttttttttttttttttttttttttttttttATCTGCAATGCCATCATC	0.4 μm
9			0.4 μm		Y152OCH	MC1R	C/A	ttttttttttttttttttttttttttttttCATCTTCTACGCACTGCGCTA	0.6 μm
10			0.4 μm		rs2228479	MC1R	G/A	ttttttttttttttttttttttttttttttttttttCTGGTGAGCGGGAGCAAC	0.375 μm
11			0.4 μm		rs1110400	MC1R	T/C	ttttttttttttttttttttttttttttttCTTCTACGCACTGCGCTACCACAGCA	
12	rs28777_F	TACTCGTGTGGGAGTTCCAT	0.4 μm	150 bp	rs28777	SLC45A2	A/C	tttttttttttttttttttttttttttttttttttttttCATGTGATCCTCACAGCAG	0.3 μm
	rs28777_R	TCTTTGATGTCCCCTTCGAT	0.4 μm						
13	Rs16891982_F	TCCAAGTTGTGCTAGACCAGA	0.4 μm	128 bp	rs16891982	SLC45A2	G/C	ttttttttttttttttttttttttttttttttttttttttttttAAACACGGAGTTGATGCA	1.2 μm
	Rs16891982_R	CGAAAGAGGAGTCGAGGTTG	0.4 μm						
14	rs12821256_F	ATGCCCAAAGGATAAGGAAT	0.4 μm	118 bp	rs12821256	KITLG	A/G	tttttttttttttttttttttttttttttttttttttttGGAGCCAAGGGCATGTTACTACGGCAC	1 μm
	rs12821256_R	GGAGCCAAGGGCATGTTACT	0.4 μm						
15	Rs4959270_F	TGAGAAATCTACCCCCACGA	0.4 μm	140 bp	rs4959270	EXOC2	C/A	tttttttttttttttttttttttttttttttttttttttttGGAACACATCCAAACTATGACACTATG	0.375 μm
	Rs4959270_R	GTGTTCTTACCCCCTGTGGA	0.4 μm						
16	rs12203592_F	AGGGCAGCTGATCTCTTCAG	0.4 μm	126 bp	rs12203592	IRF4	C/T	tttttttttttttttttttttttttttttttttttttttttttttTCCACTTTGGTGGGTAAAAGAAGG	0.3 μm
	rs12203592_R	GCTTCGTCATATGGCTAAACCT	0.4 μm						
17	rs1042602_F	CAACACCCATGTTTAACGACA	0.4 μm	124 bp	rs1042602	TYR	G/T	ttttttttttttttttttttttttttttttttttttttttttttttttttttTCAATGTCTCTCCAGATTTCA	1.25 μm
	rs1042602_R	GCTTCATGGGCAAAATCAAT	0.4 μm						
18	rs1800407_F	AAGGCTGCCTCTGTTCTACG	0.4 μm	124 bp	rs1800407	OCA2	G/A	tttttttttttttttttttttttttttttttttttttttttttttttttttttttttttttGCATACCGGCTCTCCC	0.1 μm
	rs1800407_R	CGATGAGACAGAGCATGATGA	0.4 μm						
19	rs2402130_F	ACCTGTCTCACAGTGCTGCT	0.4 μm	150 bp	rs2402130	SLC24A4	A/G	ttttttttttttttttttttttttttttttttttttttttttttttttttttttttttttTGAACCATACGGAGCCCGTG	0.75 μm
	rs2402130_R	TTCACCTCGATGACGATGAT	0.4 μm						
20	rs12913832_F	TCAACATCAGGGTAAAAATCATGT	0.4 μm	150 bp	rs12913832	HERC2	C/T	ttttttttttttttttttttttttttttttttttttttttttttttttttttttttttttttttTAGCGTGCAGAACTTGACA	1.2 μm
	rs12913832_R	GGCCCCTGATGATGATAGC	0.4 μm						
21	rs2378249_F	CGCATAACCCATCCCTCTAA	0.4 μm	136 bp	rs2378249	ASIP/PIGU	T/C	ttttttttttttttttttttttttttttttttttttttttttttttttttttttttttttttttttCCACACCTCTCCTCAGCCCA	0.18 μm
	rs2378249_R	CATTGCTTTTCAGCCCACAC	0.4 μm						
22	Rs12896399_F	CTGGCGATCCAATTCTTTGT	0.4 μm	125 bp	rs12896399	SLC24A4	T/G	tttttttttttttttttttttttttttttttttttttttttttttttttttttttttttttttttTCTTTAGGTCAGTATATTTTGGG	1.125 μm
	Rs12896399_R	GACCCTGTGTGAGACCCAGT	0.4 μm						
23	Rs1393350_F	TTTCTTTATCCCCCTGATGC	0.4 μm	124 bp	rs1393350	TYR	C/T	tttttttttttttttttttttttttttttttttttttttttttttttttttttttttttttttttttCATTTGTAAAAGACCACACAGATTT	1.1 μm
	Rs1393350_R	GGGAAGGTGAATGATAACACG	0.4 μm						
24	rs683_F	CACAAAACCACCTGTTGAA	0.4 μm	138 bp	rs683	TYRP1	T/G	ttttttttttttttttttttttttttttttttttttttttttttttttttttttttttttttttttGCTTTGAAAAGTATGCCTAGAACTTTAAT	0.175 μm
	rs683_R	TGAAAGGGTCTTCCCAGTT	0.4 μm						

**Table 2:** Information about the 17 DNA variants HIrisPlex-S (HPS) DNA test with PCR and single base extension (SBE) primer sequences and concentration. Major and minor alleles correspond to the input information entered to the HPS prediction model.

Assay Position	PCR Primers	Sequence	Concentration	Product Size	SBE Primers	Gene	HPS Model Input	Sequence	Bases	Concentration
1	rs3114908_F	CAGAACACAGCCACACCCTA	0.4 μm	118 bp	rs3114908_R	ANKRD11	C/T	TTT TTT TTT TAG AGA AGG GTC AAG CAC TT	29	0.12 μm
	rs3114908_R	CATAAAGGGGTCACCAGCAA	0.4 μm							
2	rs1800414_F	GCTGCAGGAGTCAGAAGGTT	0.4 μm	145 bp	rs1800414_R	OCA2	T/C	TTT TTT TTT TTC AGA ATC CCG TCA GAT ATC CTA	43	0.2 μm
	rs1800414_R	GGGACAAACGAATTGAGGAA	0.4 μm							
3	rs10756819_F	AAAGCAAGCTCATGTTTCCA	0.4 μm	145 bp	rs10756819_F	BNC2	A/G	TTTTTTTTTTTTGGACCAGTTATTTTGGGTTTGGA	35	1.7 μm
	rs10756819_R	CGTCATGACTAGAAAAACACCAA	0.4 μm							
4	rs2238289_F	GGAACATGAAGATTTCCCAGT	0.4 μm	112 bp	rs2238289_F	HERC2	C/T	TTT TTT TTT TTT TTT TTT TTG AGA TTG GAA GAT TGG AGC C	53	0.5 μm
	rs2238289_R	CTGATTCAGGTCTGCTGTCACT	0.4 μm							
5	rs17128291_F	CCAGCACTGCCAAAATAACA	0.4 μm	129 bp	rs17128291_R	SLC24A4	T/C	TTT TTT TTT TTT TTT TTT TTT CAA TGT GCA CTG GAT TAA AAG TC	58	1 μm
	rs17128291_R	CTCTTTGGACCCATCACCTC	0.4 μm							
6	rs6497292_F	TCTGCTGTAGAACCAATGTCC	0.4 μm	150 bp	rs6497292_R	HERC2	T/C	TTT TTT TTT TTT TTT TTT TTT TTT TTG TCT CCT GTG TCT TCA TCC T	61	0.2 μm
	rs6497292_R	GAATTGCACCTGTAGCTCCAT	0.4 μm							
7	rs1129038_F	ATGTCGACTCCTTTGCTTCG	0.4 μm	137 bp	rs1129038_F	HERC2	A/G	TTT TTT TTT TTT TTT TTT TTT TTT TTT TTT TTT TTT GAG CCA GGC AGC AGA GC	70	0.4 μm
	rs1129038_R	ACACCAGGCAGCCTACAGTC	0.4 μm							
8	rs1667394_F	CAGCTGTAGAGAGAGACTTTGAGG	0.4 μm	130 bp	rs1667394_R	HERC2	C/T	TTT TTT TTT TTT TTT TTT TTT TTT TTT TTT TTT GCA GCA ATT CAA AAC GTG CAT A	73	0.2 μm
	rs1667394_R	CACCATTAAGACGCAGCAAT	0.4 μm							
9	rs1126809_F	TGTTTCTTAGTCTGAATAACCTTTTCC	0.4 μm	100 bp	rs1126809_F	TYR	A/G	TTT TTT TTT TTT TTT TTT TTT TTT TTT TTT TTT TTT TGT ATT TTT GAG CAG TGG CTC C	77	0.05 μm
	rs1126809_R	GGTGCATTGGCTTCTGGATA	0.4 μm							
10	rs1470608_F	TTTCTTGTGTTAACTGTCCTTACAAA	0.4 μm	145 bp	rs1470608_F	OCA2	A/C	TTT TTT TTT TTT TTT TTT TTT TTT TTT TTT TTC ATT CTC TCT TAA AAA TAT TAA TTT GCA CC	62	4 μm
	rs1470608_R	GGAAAATATGTTAGGGTTGATGG	0.4 μm							
11	rs1426654_F	TTCAGCCCTTGGATTGTCTC	0.4 μm	123 bp	rs1426654_F	SLC24A5	A/G	TTT TTT TTT TTT TTT TTT TTT TTT TTT TTT TTT TTT TTT TTT TTT TGT CTC AGG ATG TTG CAG GC	86	0.16 μm
	rs1426654_R	TGAGTAAGCAAGAAGTATAAGGAGCA	0.4 μm							
12	rs6119471_F	GCAGGAGAATTGCTGGAACT	0.4 μm	170 bp	rs6119471_R	ASIP	G/C	TTT TTT TTT TTT TTT TTT TTT TTT TTT TTT TTT TTT TTT TTT TTT TGA AGG AAG AGT GAA AAT GCG TAA	91	1 μm
	rs6119471_R	AACCCGAAGGAAGAGTGAAAA	0.4 μm							
13	rs1545397_F	GGTATAGGATTATTTGGGGAATGA	0.4 μm	144 bp	rs1545397_F	OCA2	A/T	TTT TTT TTT TTT TTT TTT TTT TTT TTT TTT TTT TTT TTT TTT TTT GTA CAA CTT TGT GAA TAT ACT AAA ATA C	97	1 μm
	rs1545397_R	TGGAGATATAGAATTCACACAACATAAA	0.4 μm							
14	rs6059655_F	GTGAGGAAATCGAGGCTCAG	0.4 μm	112 bp	rs6059655_R	RALY	A/G	TTT TTT TTT TTT TTT TTT TTT TTT TTT TTT TTT TTT TTT TTT TTT TTT TTT TTT TTT TTT GCT GAT GCC CTG AGC A	76	2 μm
	rs6059655_R	AGGAGAAAGCTGCAGATCCA	0.4 μm							
15	rs12441727_F	GGGAAGAGACAGCTCCATGT	0.4 μm	137 bp	rs12441727_F	OCA2	A/G	TTT TTT TTT TTT TTT TTT TTT TTT TTT TTT TTT TTT TTT TTT TTT TTT TTT TTT TTT TTT TTT TGG CTC AGT GTG GCC TT	106	0.5 μm
	rs12441727_R	ACAATCCTGGGAGGTACACG	0.4 μm							
16	rs3212355_F	GAGTGAACCCAGGAAGATGC	0.4 μm	144 bp	rs3212355_R	MC1R	T/C	TTT TTT TTT TTT TTT TTT TTT TTT TTT TTT TTT TTT TTT TTT TTT TTT TTT TTT TTT TTT TTT TTT TTT TCC GAA GCC CAG CAG G	113	1.5 μm
	rs3212355_R	CATCAAAGGCAGACCTCTCG	0.4 μm							
17	rs8051733_F	AGGCGGTGGTCTCTCTCTC	0.4 μm	124 bp	rs8051733_R	DEF8	T/C	TTT TTT TTT TTT TTT TTT TTT TTT TTT TTT TTT TTT TTT TTT TTT TTT TTT TTT TTT TTT TTT TTT TTT TTC ACC CTG CCT GTC TCG	115	1.6 μm
	rs8051733_R	TTGCAACAGGAGGGTCTAGG	0.4 μm							

**Table 3:** Summary of the skeletal biological profile analysis for each individual. Population affinity was estimated using Fordisc, biological sex estimation was conducted using MorphoPASSE and age at death was estimated using ADBOU. Additionally, the known population affinity, sex and age of the individuals are presented.

Individual	Population Affinity Estimation	Known Population Affinity	Sex Estimation	Known Sex	Age Interval	Age Point Estimate	Known Chronological Age
1	White	White	Male	Male	35–75	49.8	60
2	White	White	Male	Male	>50	79.2	88
3	White	White	Male	Male	30–75	48.1	42
4	White	White	Female	Female	30–65	41.2	66
5	White	White	Male	Male	45–85	63.7	62

**Table 4:** Phenotyping predictions according to the HIrisPlex-S online tool.

	Phenotypic Characteristics		Individuals				
			1	2	3	4	5
Eye	Eye Color	Blue eye	0.926	0.067	0.026	0.948	0.848
		Intermediate	0.057	0.13	0.063	0.038	0.088
		Brown eye	0.017	0.803	0.912	0.014	0.065
Hair Color	Hair Color	Blond hair	0	0	0	0	0
		Brown hair	0	0.006	0.006	0.001	0
		Red hair	1	0.994	0.994	0.999	1
		Black hair	0	0	0	0	0
Hair Shade		Light hair	0.936	0.071	0.367	0.973	0.998
		Dark hair	0.064	0.929	0.633	0.027	0.002
Skin	Skin Color	Very pale skin	0.231	0.074	0.004	0.029	0.0421
		Pale skin	0.711	0.442	0.079	0.675	0.51
		Intermediate skin	0.058	0.479	0.692	0.293	0.069
		Dark skin	0	0.005	0.2	0.003	0
		Dark to black skin	0	0	0.025	0	0

## Data Availability

Data is available upon request to the authors.
